# Psoriatic Arthritis and Nail Psoriasis in a Patient with Concomitant Atopic Dermatitis

**DOI:** 10.1155/2018/4125856

**Published:** 2018-02-18

**Authors:** Gerhard Eichhoff, Noriko Soffi Harun

**Affiliations:** ^1^Wellington Regional Hospital, Private Bag 7902, Wellington 6242, New Zealand; ^2^Department of Rheumatology, Hutt Hospital, Lower Hutt, New Zealand

## Abstract

Coincidence of psoriasis and atopic dermatitis (AD) is considered to be very rare, as a result of the different underlying immunopathology. This case report describes a patient with long history of atopy and AD who developed psoriatic nail changes and psoriatic arthritis (PsA). The patient's skin, however, revealed only eczematous lesions without manifestation of psoriasis.

## 1. Introduction

Psoriasis and atopic dermatitis (AD) are the most frequent chronic inflammatory skin diseases. Both are estimated to have prevalence of 2–5% in the adult population. These skin diseases are associated with other immune disorders, such as psoriatic arthritis or allergic asthma [1]. Approximately 20% of patients with psoriasis develop psoriatic arthritis (PsA), whereas 5% of patients with PsA have no active skin lesions [2]. PsA is correlated with psoriatic nail changes [3].

While both psoriasis and AD are common skin disorders, concomitant occurrence of these two diseases is rare. This rarity is likely explained by recent immunologic studies that revealed that psoriasis is a more TH1/TH17-driven disease, whereas AD is a predominantly TH2-driven disease [4].

Here, the authors report the case of a patient with PsA in the hands, psoriatic nail changes without accompanying psoriasis of the skin, and concomitant active AD.

## 2. Case Report

A 33-year-old male patient of European descent was referred to the dermatological outpatient clinic for recurrent paronychia that did not respond to several courses of terbinafine. The patient had a history of atopy with hay fever and AD, which manifested in early childhood. In his early 20s, his AD was treated with a one-year course of cyclosporine. Since then, the eczema was managed with topical steroids. He had no history or family history of psoriasis or PsA.

**Figure 1:**
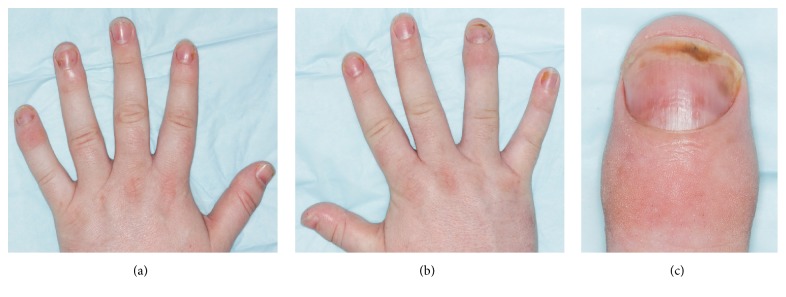
Swelling with redness of the distal interphalangeal (DIP) joints of the left (a) and right (b) hand. Predominantly the right index and left little finger are affected. Psoriatic nail changes with oil drops and distal onycholysis occurred on almost all fingernails. (c) Close-up view of DIP on the right hand.

Over the prior 12 months, he developed painful swelling in his joints in his hands and concomitant nail changes. Since onset of these symptoms, the patient did not notice major changes in the severity of his eczema that occurred with regular most likely stress-related minor flare-ups. The patient presented with oil drops on almost all fingernails and on nails of both big toes together with mild distal onycholysis. Furthermore, swelling with redness of the distal interphalangeal joints (DIP) of the right index and left little finger was evident (Figure 1). These findings were consistent with psoriatic nail changes and PsA. An X-ray of the patient's hands showed entheseal expansion of the DIP of the right ring finger and left little finger, suggesting PsA. Therefore, PsA was diagnosed as the patient met 4 points of the classification criteria for psoriatic arthritis (CASPAR) [5].

**Figure 2:**
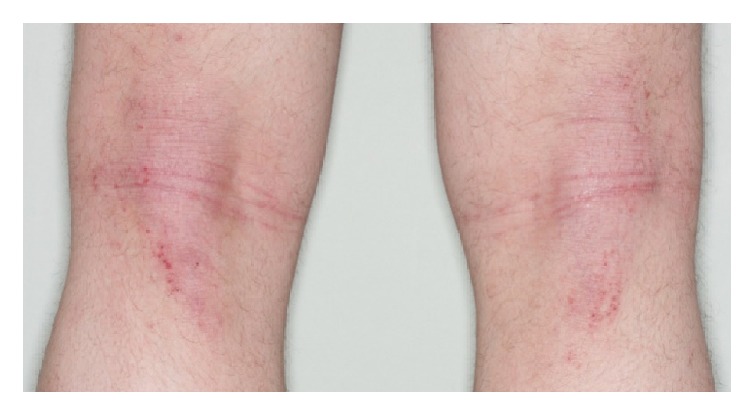
Ill-defined erythematous patches on the backs of the knees with scaling, lichenification, and excoriations.

Whole-body examination revealed active AD on the backs of the knees with ill-defined erythematous patches with scaling, lichenification, and excoriations (Figure 2). Besides the nail changes, no psoriatic skin lesions were observed. Laboratory analyses underscored the atopy of the patient with elevated IgE levels (689 IU/mL). Rheumatoid factor was not detectable, and C-reactive protein was in the normal range. Treatment with methotrexate 15 mg once weekly was commenced. The patient was recommended to continue Mometasone Furoate ointment for his AD. At 8 weeks of follow-up, neither the patient's joint pain and swelling nor nail changes improved markedly. His eczema did, however, slightly improve. It is yet too early to judge the efficacy of methotrexate.

## 3. Discussion

Concomitant psoriasis and AD are rare. Henseler and Christophers found that AD is 25 times less frequently associated with psoriasis than one would expect based on prevalence of the individual diseases [6]. In 1987, Christophers and Henseler hypothesized that psoriasis and AD were mutually exclusive [7]. In recent years, immunological studies provided support for this hypothesis, attributing this exclusion to the more TH1/TH17-dominated immune response underlying psoriasis and the TH2-dominated immune response found in patients with atopic eczema. Furthermore, independent regulations of these two immune phenotypes have been shown to rarely overlap [4].

On the other hand, AD and psoriasis can share both clinical and immunological features. This especially applies to subtypes of AD, such as AD within Asian populations, paediatric AD, and intrinsic AD, where TH17 does seem to play a key role [8]. The patient reported here, being from European descent and suffering from early-onset extrinsic eczema, does not fit into these subtypes of AD.

The IL-23/IL-17 axis plays a crucial role not only in the pathogenesis in psoriasis but also in PsA [9]. Nail changes are a marker of psoriatic arthritis, and magnetic resonance imaging showed that nail enthesis is associated with the nail, the distal phalanx, and distal interphalangeal joints and therefore may cause PsA [10].

This report provides the first description to date of a patient with AD who developed PsA with nail changes without further skin manifestation of psoriasis. Whereas no other study on concomitant psoriasis and AD described patients with PsA, Hajdarbegovic et al. reported PsA to be significantly less likely to be associated with atopy than isolated skin psoriasis [11].

Treatment of patients with concomitant psoriasis and AD can be complicated by the fact that tumor necrosis factor-alpha (TNF-alpha) inhibitors and ustekinumab, the monoclonal antibody against the p40 subunit of interleukin-12 (IL-12) and IL-23, have been shown to provoke eczema or a flare of AD as a side effect [12]. As a result, systemic treatment with cyclosporine, methotrexate, and/or apremilast is preferable as these treatments can be efficacious for both AD and psoriasis, including PsA [13]. Further research of the involved cytokines including the patient's individual cytokine profile will be needed to offer these patients more targeted treatment.
